# Analysis and improvement of human-induced vibration comfort of articulated steel pedestrian bridges

**DOI:** 10.1038/s41598-023-44456-1

**Published:** 2023-10-11

**Authors:** Shuo Yang, Xiaojun Ning

**Affiliations:** Faculty of Civil Engineering and Mechanics, Kunming University of Science and Technology, Kunming, 650500 Yunnan China

**Keywords:** Civil engineering, Computational methods

## Abstract

The effect of different pedestrian densities (0.2, 0.5, 1, 1.5 and 4.6 persons/m^2^) on a pedestrian bridge is studied, the comfort level is evaluated according to the acceleration peak, and the most sensitive part of the acceleration response employs tuned mass dampers (TMDs) for vibration control. The study shows that the bearing capacity level of the pedestrian bridge with articulated piers meets the standard. Compared with a pier rigid connection system, the structural dynamic characteristics of pier articulation do not change much, and the vertical frequency and peak acceleration in the span are slightly smaller. The comfort evaluation results of the bridge with articulated piers are the same as those of the bridge with a rigid pier. The TMD setting can effectively reduce the human-induced vibration time response, the vibration reduction efficiency can reach 52%, and the comfort level changes from CL2 to CL1 after vibration reduction.

## Introduction

Pedestrian bridges are usually designed to be narrow and long due to the special nature of their service recipients, and their fundamental frequencies are often much lower than those of vehicular bridges with similar structural forms. These bridges are prone to excite large vibrations under pedestrian excitation, which may lead to pedestrian psychological discomfort and even dizziness in severe cases. Therefore, it is necessary to study the comfort of pedestrian bridge human-induced vibration.

Pedestrian bridges are designed to serve mainly pedestrians, and their design loads are mainly crowd loads. The design load is a mathematical model used to describe the static and dynamic action of a crowd on a bridge structure with a specific reliability guarantee rate, which is determined during the design reference period of the bridge structure.

In addition to the consideration of load carrying capacity, the normal serviceability of the bridge, such as deflection and crack width, must also be included in the structural design. Compared to other types of bridges, the normal use of dedicated pedestrian bridges must also include human-induced vibration comfort or assessment.

In 1996, China released the Technical Specification for Urban Footbridges and Pedestrian Walkways (CJJ69-95)^1^, which for the first time, presented the requirements for the comfort of footbridges, stipulating that the vertical self-oscillation frequency of footbridges should not be less than 3 Hz. This regulation plays an important role in ensuring the safety and comfort of pedestrians. In addition, for the structural design of buildings, China has issued a number of technical specifications. For example, the Technical Regulations for Concrete Structures of High-rise Buildings (JGJ3-2010)^2^ and Technical Regulations for Steel Structure of High-rise Civil Buildings (JGJ99-2015)^3^ both suggest that the vertical vibration frequency of a building cover structure should not be less than 3 Hz. In addition, these specifications also describe the vertical vibration acceleration limits of the building cover under different vertical self-oscillation frequencies. For concrete structures, the Code for the Design of Concrete Structures (GB50010-2010)^4^ also specifies the limits of vertical self-oscillation frequencies for concrete building covers of different use functions. In addition to Chinese codes, other countries have developed specific standards for the vibration comfort of building covers and pedestrian bridges. For example, the European norm^5^, British Specification (BS5400)^6^ and German norm (EN03)^7^ all specify the vertical self-oscillation frequency and vibration acceleration of the structure and other indicators. The German code (EN03) specifies the vibration acceleration limits for different structure types, including building covers, bridges and other structure types.

In recent years, with the continuous development and improvement of construction technology in China, the vibration comfort standards of building covers and pedestrian bridges have been upgraded and improved. For example, the Building Vibration Load Standard (GB/T51228-2017)^8^ provides detailed regulations for vibration loads on buildings, including elements such as load modes and frequency ranges. Meanwhile, the Technical Standard for Vibration Comfort of Building Cover Structure (JGJ/T441-2019)^9^ specifies the vertical self-oscillation frequency, vibration acceleration and comfort index of building covers, taking into account factors such as human sensitivity and vibration frequency. In addition, the implementation of the yet-to-be-released "urban footbridge and footpath technical specifications (draft for comment)" (CJJ/69-20XX)^10^ also provides for the vibration comfort of pedestrian bridges, including self-vibration frequency, vibration acceleration limits and damping ratio values and other elements. The introduction of these specifications has provided important technical guidance and assurance for the design, construction and use of building covers and footbridges in China.

To grasp the vibration characteristics of a pedestrian bridge with an articulated pier steel structure under pedestrian load, the finite element software MIDAS civil is used to carry out the comfort analysis of a pedestrian bridge under different pedestrian density conditions, evaluate the comfort level according to the vibration acceleration, and control the vibration by setting tuned mass dampers (TMDs) for the parts that do not meet the comfort requirements.

## Project overview

**Figure 1:**
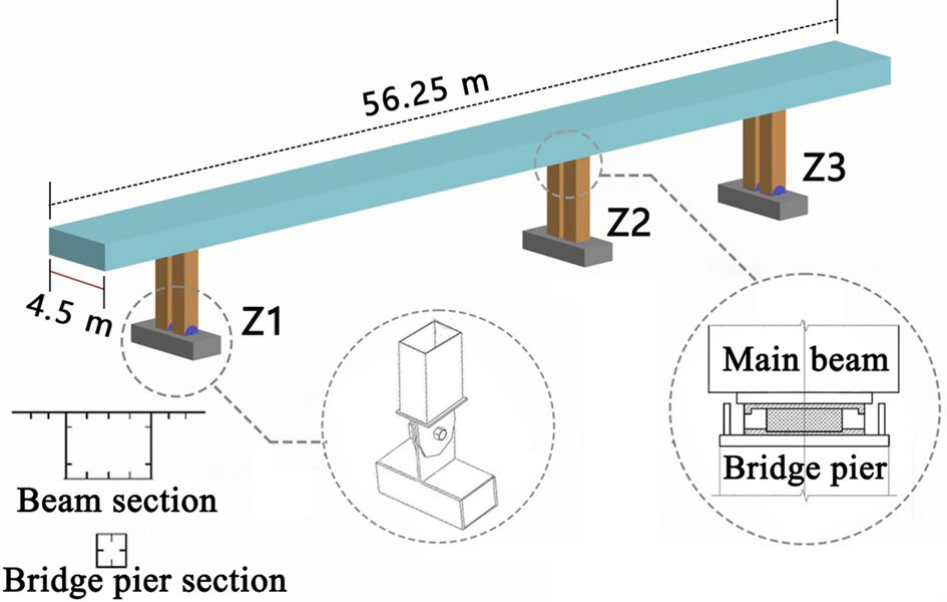
Steel pedestrian bridge with hinged piers.

The object of this study is a steel pedestrian bridge with hinged piers. As the name suggests, it is a form of bridge in which pin bearings are set between the bottom of the piers and the bearings of the bridge substructure so that the set piers can be rotated in the longitudinal direction, as shown in Fig. 1.

The span combination of the whole bridge is 3.25 m + 37 m (first span) + 13 m (second span) + 3 m, the total length of the bridge is 56.25 m, and the area of the bridge deck is 253.125 m2. The Z1 pier and Z3 pier are rigidly connected to the main beam, and pin bearings are set between the pier bottom and the bearing platform. The Z2 pier is set in the Weishan Road side parting zone, the Z2 pier top set is a GBZY350 × 63 plate rubber bearing, and the main bridge box girder is connected. The second phase of constant load: considering the bridge deck pavement, the railing load is 4.6 kN/m.

## Static load test

To study the deformation and stress values of the bridge structure under the static load of a crowd on a continuous rigid pedestrian bridge with hinged piers, it is necessary to understand whether the actual performance of the structure and the indices of stiffness and strength of the structure are in accordance with the expected design values. The main span and the second span of the continuous rigid pedestrian bridge with articulated piers were selected as the test span for the static load test. Through the arrangement of a certain number of measurement points, a more comprehensive analysis of the structure of the force can be performed, and the quality of design and construction can be tested to understand the engineering structural performance, safety and reliability degree to judge the actual load-bearing capacity of the bridge structure.

**Figure 2:**
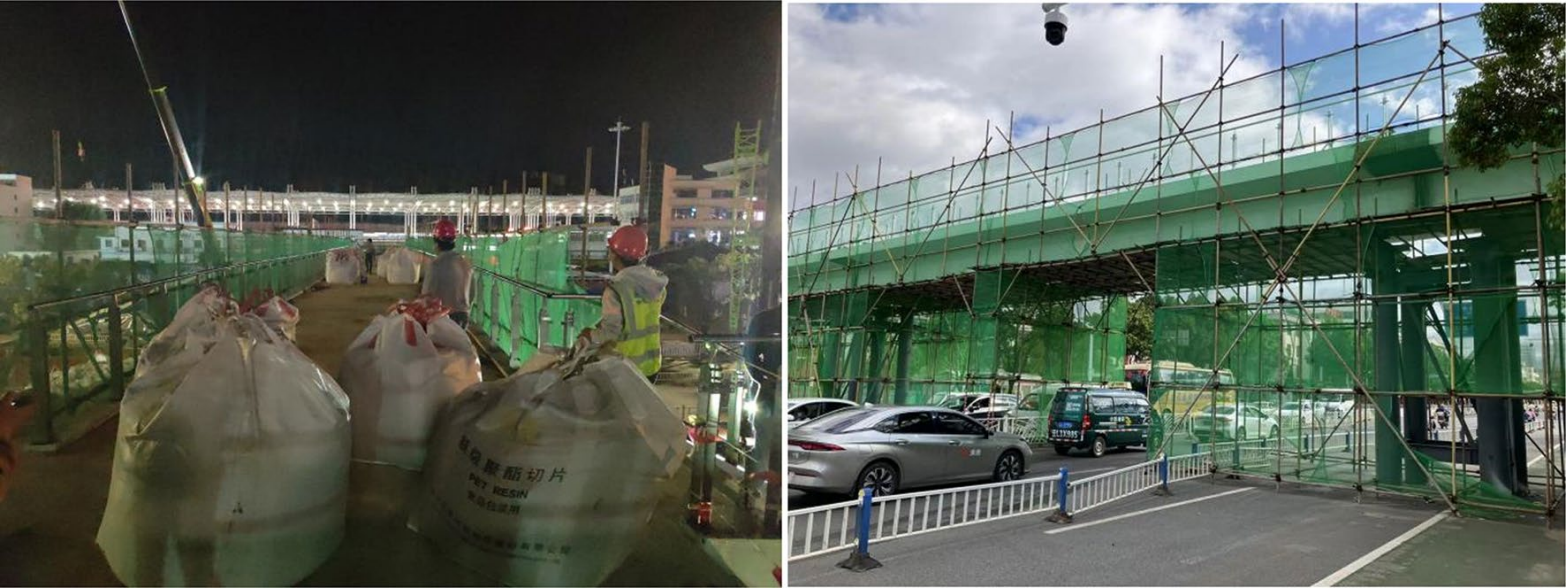
Test site and sensors.

**Figure 3:**
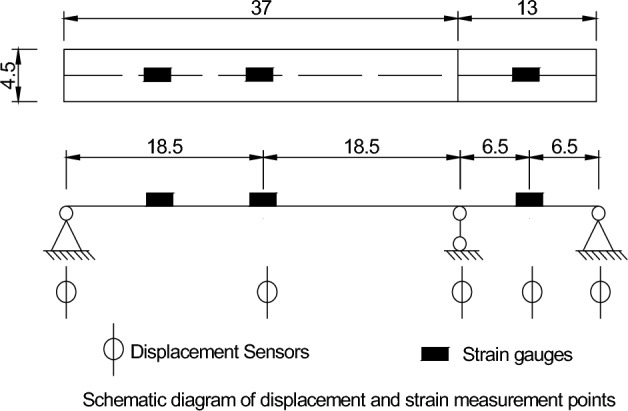
Schematic diagram of the displacement.

The design variable load of this bridge is a crowd load, and the longitudinal most unfavourable distribution of the bridge structure is carried out according to the above design load. The maximum internal force value (bending moment) of the control section under the design load and the test load are calculated, and the larger value is taken as the basis for the test loading. According to the bridge design and construction drawings, the bridge phase II constant load: bridge deck pavement and railing load is taken as 4.6 kN/m (both sides). Referring to the specification "Technical Specification for Urban Pedestrian Bridges and Pedestrian Walkways" (CJJ 69-95), the unified value of the crowd load is 4.2 kPa or 16.80 kN/m. The test load required during the test is applied by counterweight sandbags, as shown in Fig. 2. Strain gauge measurement points were laid out, and displacement gauges were set up in the span and at the bearing locations, as shown in Fig. 3.

According to the structure of the bridge, the force characteristics were determined according to the specification requirements test using graded loading. Based on the calculation and analysis, a total of 2 static load test conditions were set up in the main span and the second 1 span, and the loading positions of the test conditions are shown in Table 1. The order in which operating conditions 1, 2 and 3 are performed does not affect the results.

**Table 1:** Static loading conditions.

Test conditions	Load location	Load (KN/m)	Duration (min)	Resting time (min)
Condition 1	Main span	16.80	10	30
Condition 2	Sub-straddle	16.80	10	30
Condition 3	Whole bridge	16.80	10	30

No significant deformation of the bridge members occurred during the loading process. The test results are shown in Table 2. The Technical Specification for Urban Footbridges and Pedestrian Walkways" (CJJ 69-1995), is Article 2.5.2, "Superstructure, the maximum vertical deflection calculated by the pedestrian load, beam-slab type bridge shall not exceed L/600." According to the calculated span diameter of the mid-span, L/600 = 61.67 mm. After data summary, the maximum displacement of the three working conditions is 17.62 mm, which does not exceed the specification limit, and the strength meets the design requirements. In order to further verify the deflection of this bridge, Midas civil finite element software was used for modeling, and the same working conditions were set with the field test. The results show that the maximum deflection occurs in condition 1 with a value of 18.25 mm. The error is only 3.58%, which indicates that the experimental results are correct.

**Table 2:** Static test results.

Test conditions	Maximum displacement value (m)	Relative residual displacement (%)	Maximum displacement position	Maximum strain (με)	Relative residual strain (%)	Maximum strain position
Condition 1	0.01762	− 4.37	Span centre of the main span	115	0	Span centre of the main span
Condition 2	0.00045	− 1.74	Span centre of the main span	21	− 0.16	Span centre of the secondary span
Condition 3	0.01732	− 1.60	Span centre of the main span	134	0	Span centre of the main span

## Pedestrian load model

According to a large number of statistical results^11^, the continuous foot loading of pedestrians is expanded according to Fourier series. The first- and second-order harmonic frequencies of vertical loading are in the range of 1.25–4.6 Hz, and the first-order harmonic frequencies of lateral loading are in the range of 0.5–1.2 Hz. When the fundamental frequency of the pedestrian bridge is in the stated range, a resonance occurs between pedestrians and the bridge, thus causing discomfort to pedestrians. There is no example to date of a large vibration of a real bridge caused by a second-order harmonic load on a pedestrian vertical walk^12^. Based on a combination of domestic and international industry standards and research results^13^, only vertical walking first-order harmonic pedestrian loads are considered in this paper. German EN03 proposes that the frequency range of the vertical comfort examination of flyover is 1.25–3 Hz and that the frequency range of lateral comfort examination is 0.5–1.2 Hz.

The Technical Specification for Urban Footbridges and Pedestrian Underpasses (Draft for Comments) (CJJ/69-20XX), which has not yet been released and implemented in China, proposes a corresponding vertical pedestrian vibration load excitation formula for the pedestrian loads of footbridges. In the human-caused vibration comfort analysis, the frequency range for the vertical comfort of the flyover is the same as that of the German code, and it is stipulated that the pedestrian density should not be greater than 1.5 persons/m^2^ or less than 1.0 persons/m^2^. The specification for the crowd density requirements is taken as d = 0.2, 0.5, 1, 1.5, and 4.6 persons/m^2^. However, for d = 4.6, the crowd cannot move but can be in place.

The most comprehensive definition of comfort is described in the German standard EN03, which not only considers the firm load and lateral load but also selects different load patterns according to different population densities. The same equations for the pedestrian simple harmonic loads are given in the codes of both countries, and the normalization methods involved in the following are calculated using the load expressions given in the German code EN03. The human-induced vibration simple harmonic loads are given in Eq. (1).1

Here, $\mathrm{p}(\mathrm{t})$ is the pedestrian vibration load per unit area $\left(N/{\mathrm{m}}^{2}\right).\mathrm{ P}\cdot \mathrm{cos}(2\pi \mathrm{fst})$ represents the harmonic load during single person walking $(\mathrm{N})$; $\mathrm{P}$ is the fraction of force generated by a single pedestrian walking with step frequency ${\mathrm{f}}_{\mathrm{s}} (\mathrm{N})$; ${\mathrm{f}}_{\mathrm{s}}$ is the walking frequency, which is generally assumed to be the inherent frequency of the flyover; ${\mathrm{n}}^{\mathrm{^{\prime}}}$ is the equivalent number of pedestrian flows when the loading area is S; $\psi$ is the discount factor when the dropout frequency is close to the inherent frequency of the structure; $\xi$ is the damping ratio of the structure; $\mathrm{n}$ is the number of rows when the loading area is S; and $\mathrm{n}=\mathrm{S}\times \mathrm{d}$. When the direction of human-induced vibration is firm, $\mathrm{P}$ is taken as 280 $(\mathrm{N})$.

For the discount factor $\psi$, the current "Building Vibration Load Standard" in China^8^ has a clear range of values for the load reduction factor of pedestrian bridges, which is 0.25. For the damping ratio of the structure, the German code and the Chinese "Building Vibration Load Standard" take the same value, $0.4\mathrm{\%}$.

## Pier articulation and rigid connection of the modal comparison analysis

The 40th-order modal analysis was performed by using the block Lanczos method, and the first 3-order vibration patterns and modal frequencies were extracted after the analysis, i.e., 3.92 Hz for 1st-order symmetric vertical bending, 10.79 Hz for 2nd-order torsion and 20.48 Hz for 3rd-order antisymmetric vertical bending in the articulated mode. The first-order symmetrical vertical bending at 3.96 Hz, the second-order torsion at 10.95 Hz and the third-order antisymmetrical vertical bending at 20.51 Hz for the rigid connection mode are shown in Table 3.

**Table 3:** Comparison of natural frequencies.

Frequency	Pier articulation	Pier rigid connection
1st-order natural frequency	3.92	3.96
2nd-order natural frequency	10.79	10.95
3rd-order natural frequency	20.48	20.51

**Figure 4:**
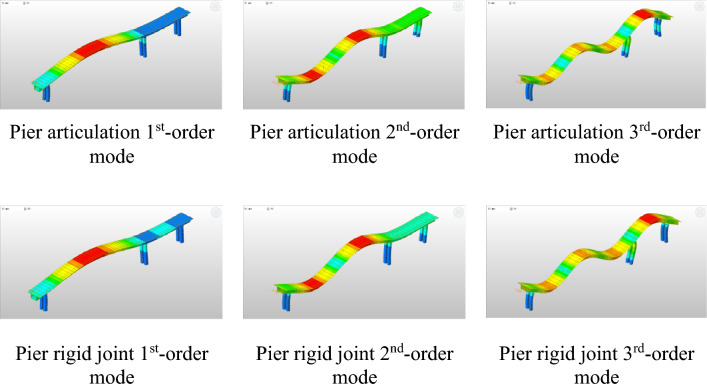
Comparison of articulated and rigid joints in the first three orders of modalities.

From Table 3 and Fig. 4, it can be seen that the pier articulation and rigid mode are close to each other, and the difference in the first three order frequencies is also very small. The frequency of the pier rigid joints is slightly greater than that of the articulated joints.

## Comfort analysis

Vertical comfort analysis is carried out by applying a pedestrian load according to the vibration pattern of the 1st-order mode. According to the loading position, there are three working conditions: Condition 1 applies the load in the main span, Condition 2 applies the load in the secondary span, and Condition 3 applies the load in both the main and secondary spans. The German Pedestrian Bridge Design Guide EN03 incorporates the latest research results since 2000 and covers the avoidance of the sensitive frequency method and the limitation of the dynamic response method, which has obvious superiority compared with other codes. In addition, EN03 takes into account the different pedestrian densities on the bridge and the different pedestrian vibration perception expectations, and its comfort level is divided according to the different pedestrian densities. The comfort evaluation criteria are shown in Table 4.

**Table 4:** Comfort grading in the German EN03 design guidelines.

Specification	Comfort level	Comfort	Maximum vertical acceleration	Lateral acceleration max
EN03	CL1	Best	< 0.50 m/s^2^	< 0.10 m/s^2^
	CL2	Moderate	0.50–1.00 m/s^2^	0.10–0.30 m/s^2^
	CL3	Worst	1.00–2.50 m/s^2^	0.30–0.80 m/s^2^
	CL4	Unacceptable	> 2.50 m/s^2^	> 0.80 m/s^2^

### Articulation comfort analysis

In MIDAS civil, a "Nodal Dynamic Load" is used to apply a pedestrian load on the surface of the main beam with a 1 m spacing between each node and a 50 s analysis time. Based on the vertical first-order frequency of the pedestrian bridge, the pedestrian load functions can be calculated for different working conditions and pedestrian densities. When the Z1 and Z3 piers are articulated, the maximum acceleration response points of the main span and secondary span are located at the middle of the span of the main span under the three working conditions. The maximum articulated acceleration and comfort evaluation are shown in Table 5.

**Table 5:** Articulated acceleration maximum and comfort evaluation.

Work conditions	d (person/m^2^)	Maximum value acceleration (m/s^2^)		Comfort level
		21 (+)	21 (−)	
1	0.2	0.333	− 0.333	CL1
	0.5	0.563	− 0.563	CL2
	1	2.163	− 2.163	CL3
	1.5	2.649	− 2.649	CL4
	4.6	4.638	− 4.639	CL4
2	0.2	0.002	− 0.002	CL1
	0.5	0.003	− 0.003	CL1
	1	0.012	− 0.012	CL1
	1.5	0.015	− 0.015	CL1
	4.6	0.026	− 0.026	CL1
3	0.2	0.335	− 0.335	CL1
	0.5	0.566	− 0.566	CL2
	1	2.175	− 2.175	CL3
	1.5	2.664	− 2.664	CL4
	4.6	4.665	− 4.666	CL4

**Figure 5:**
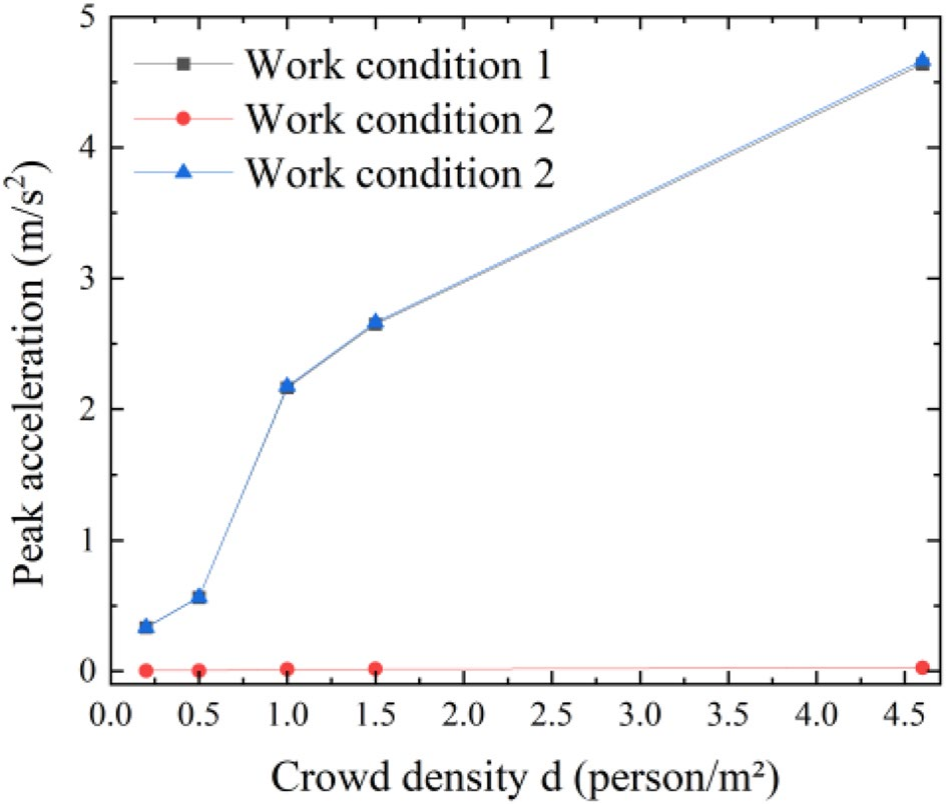
Articulated acceleration response maxima versus pedestrian density.

From Table 5 and Fig. 5, when the piers are articulated, the maximum values of acceleration are close to when pedestrian loads are applied to the main span (Case 1) and the full bridge (Case 3). When pedestrian loads are applied at the subspan, the maximum value of midspan acceleration is extremely small and does not excessively affect the structural response. The acceleration response produced by Case 3 is higher than that of Case 1 for the same pedestrian density. The maximum value of acceleration in Condition 3 is the sum of the acceleration values in Conditions 1 and 2. In Case 1 and Case 3, when the crowd density d ≥ 0.5 person/m^2^, the maximum acceleration already exceeds 0.5 m/s^2^, which does not meet the CL1 comfort level. Therefore, vibration damping measures need to be applied to the structure.

**Figure 6:**
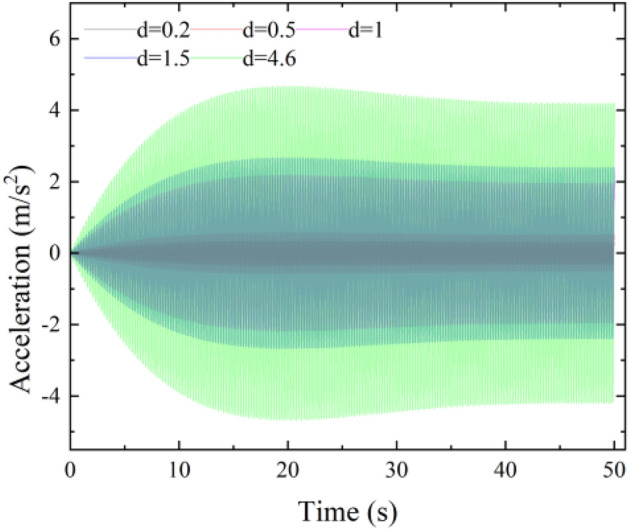
Typical time course curve of articulated Condition 3.

The typical time course curves of the midspan acceleration response with time for the main span under working Condition 3 when the pedestrian density is 0.5, 1, 1.5, and 4.6 persons/m^2^ are shown in Fig. 6. As seen from Fig. 5, the acceleration response becomes larger as the loading time becomes longer and finally gradually stabilizes.

### Rigid joint comfort analysis

When the Z1 and Z3 piers are just connected, the maximum point of the acceleration response of the main span and secondary span is located at the middle of the main span under the three working conditions. The maximum values of rigid joint acceleration and comfort evaluation are shown in Table 6.

**Table 6:** Maximum values of the rigid joint acceleration and comfort evaluation.

Work conditions	d (person/m^2^)	Maximum value acceleration (m/s^2^)		Comfort level
		21 (+)	21 (−)	
1	0.2	0.340	− 0.340	CL1
	0.5	0.575	− 0.575	CL2
	1	2.208	− 2.207	CL3
	1.5	2.704	− 2.703	CL4
	4.6	4.736	− 4.734	CL4
2	0.2	0.002	− 0.002	CL1
	0.5	0.003	− 0.003	CL1
	1	0.013	− 0.013	CL1
	1.5	0.016	− 0.016	CL1
	4.6	0.028	− 0.028	CL1
3	0.2	0.342	− 0.342	CL1
	0.5	0.579	− 0.578	CL2
	1	2.220	− 2.220	CL3
	1.5	2.721	− 2.720	CL4
	4.6	4.764	− 4.763	CL4

**Figure 7:**
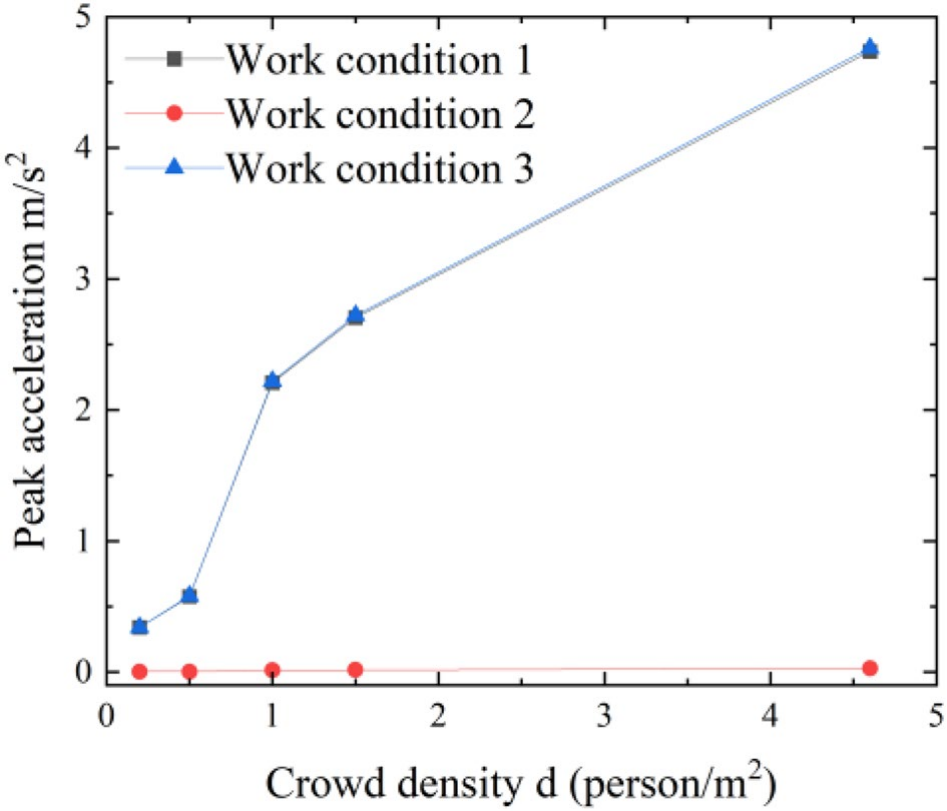
Maximum value of the rigid joint acceleration response versus pedestrian density.

From Table 6 and Fig. 7, it can be seen that the performance of the pier rigid and pier hinged joints is close in all operating conditions; the comfort rating is exactly the same. However, it can be seen that the maximum acceleration of the rigid joint is larger than that of the articulated joint in all operating conditions, and the dynamic response of the structure is more significant.

**Figure 8:**
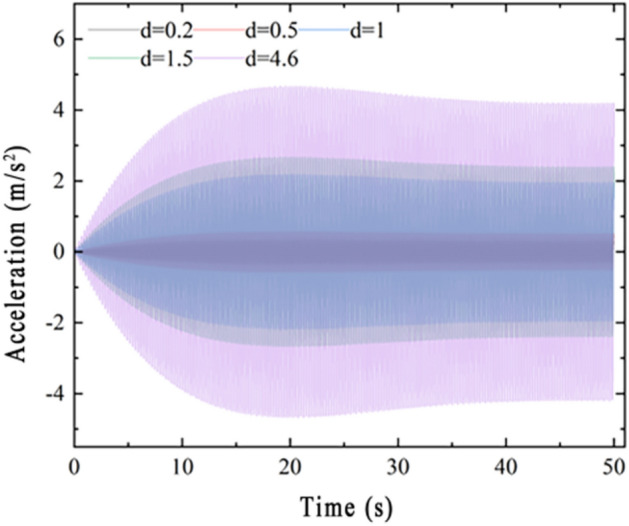
Typical time course curve of rigid connection Condition 3.

Figure 8 shows the typical time course curves of the midspan acceleration response of the main span with time for pedestrian densities of 0.5, 1, 1.5, and 4.6 persons/m^2^ under working Condition 3. As seen from Fig. 5, the acceleration response becomes larger as the loading time becomes longer and finally gradually stabilizes.

## TMD vibration damping analysis

A tuned mass damper (TMD) is an energy dissipation and damping device commonly used in real structures. It consists mainly of mass blocks, springs and dampers and is generally supported or suspended from the structure. By adjusting the mass or stiffness of the damper system, the inherent frequency of the system is changed so that its frequency is close to the inherent frequency of the structure under control. When the main structure vibrates, the TMD system generates an inertial force applied to the structure in opposition to the structure itself, thus achieving the purpose of controlling the vibration response of the structure.

At present, the optimal parameter calculation formula for TMD control of an undamped structural system (main structure damping c = 0) is commonly used in engineering. By selecting the optimum damping ratio and the optimum frequency ratio, the vibration response of the main system will be minimized when the main system and the damper are resonating. When considering structural damping, Hoang et al.^14^ used the numerical solution method to propose the formulation of the optimal parameters of a TMD system with damped structures for different optimization objectives under sinusoidal loads. These two different sets of parameter optimization formulas are also applicable in the case of external loads in the form of sinusoidal loads. When the mass ratio of the TMD and main structure is in the range of 3–4% and the damping ratio of the main structure is in the range of 0–0.15, the error of the calculated optimal parameters of the TMD does not exceed 1%. In general, the greater the mass is, the better the damping effect. However, the mass of the TMD cannot be increased indefinitely; it is too difficult to construct when the mass is too large and can adversely affect the main structure. Therefore, a mass of the damping device between 1 and 5% of the modal mass of the main structure vibration pattern is more appropriate^15^.

According to the above, when pedestrian loads are simultaneously applied to the main and secondary spans of the flyover and the pedestrian density is greater than 0.5 persons, the maximum value of the acceleration response is exceeded, and vibration damping control is needed. When selecting the TMD parameters, the optimal damping ratio ${\upxi }_{\mathrm{opt}}$ and the optimal frequency ratio ${\mathrm{f}}_{\mathrm{opt}}$ calculated from the mass ratio $\upmu$ can be used to further determine the spring stiffness ${\mathrm{K}}_{\mathrm{d}}$ and the damping coefficient ${\mathrm{C}}_{\mathrm{d}}$ of the TMD. $\mathrm{Each parameter of the TMD is determined by the following equation}$^16^:

$$\begin{aligned} & {\text{m}}_{{\text{d}}} = {\mu M}_{{\text{i}}} \\ & {\upxi }_{{{\text{opt}}}} = \sqrt {\frac{{3{\upmu }}}{{8(1 + {\upmu })^{3} }}} \\ & {\text{f}}_{{{\text{opt}}}} = \frac{1}{{1 + {\upmu }}} \\ & {\text{f}}_{{\text{d}}} = {\text{f}}_{{{\text{opt}}}} {\text{f}}_{{\text{i}}} \\ & {\text{K}}_{{\text{d}}} = \left( {2{\pi f}_{{\text{d}}} } \right)^{2} {\text{m}}_{{\text{d}}} \\ & {\text{C}}_{{\text{d}}} = 2 \cdot \left( {2{\pi f}_{{\text{d}}} } \right){\text{m}}_{{\text{d}}} {\upxi }_{{{\text{opt}}}} \\ \end{aligned}$$

Here, ${\mathrm{m}}_{\mathrm{d}}$ is the TMD mass; ${\mathrm{M}}_{\mathrm{i}}$ is the ith order vertical modal mass of the structure; ${\mathrm{f}}_{\mathrm{d}}$ is the optimized frequency; and ${\mathrm{f}}_{\mathrm{i}}$ is the ith order modal frequency of the structure.

For the articulated pier and column footbridge, when the mass ratio $\upmu$ is taken as 0.03, the parameters are shown in Table 7.

**Table 7:** TMD parameters.

${\upmu }$	${\text{M}}_{{\text{i}}}$/$\left( {{\text{kg}}} \right)$	${\text{m}}_{{\text{d}}}$/$\left( {{\text{kg}}} \right)$	${\upxi }_{{{\text{opt}}}}$	${\text{f}}_{{{\text{opt}}}}$	${\text{f}}_{1}$/$\left( {{\text{Hz}}} \right)$	${\text{f}}_{{\text{d}}}$/$\left( {{\text{Hz}}} \right)$	${\text{K}}_{{\text{d}}} { }\left( {{\text{N}}/{\text{m}}} \right)$	${\text{C}}_{{\text{d}}}$(N s/m)
0.03	3496	104.88	0.1	0.97	3.92	3.8024	59,864.2	500.89

**Figure 9:**
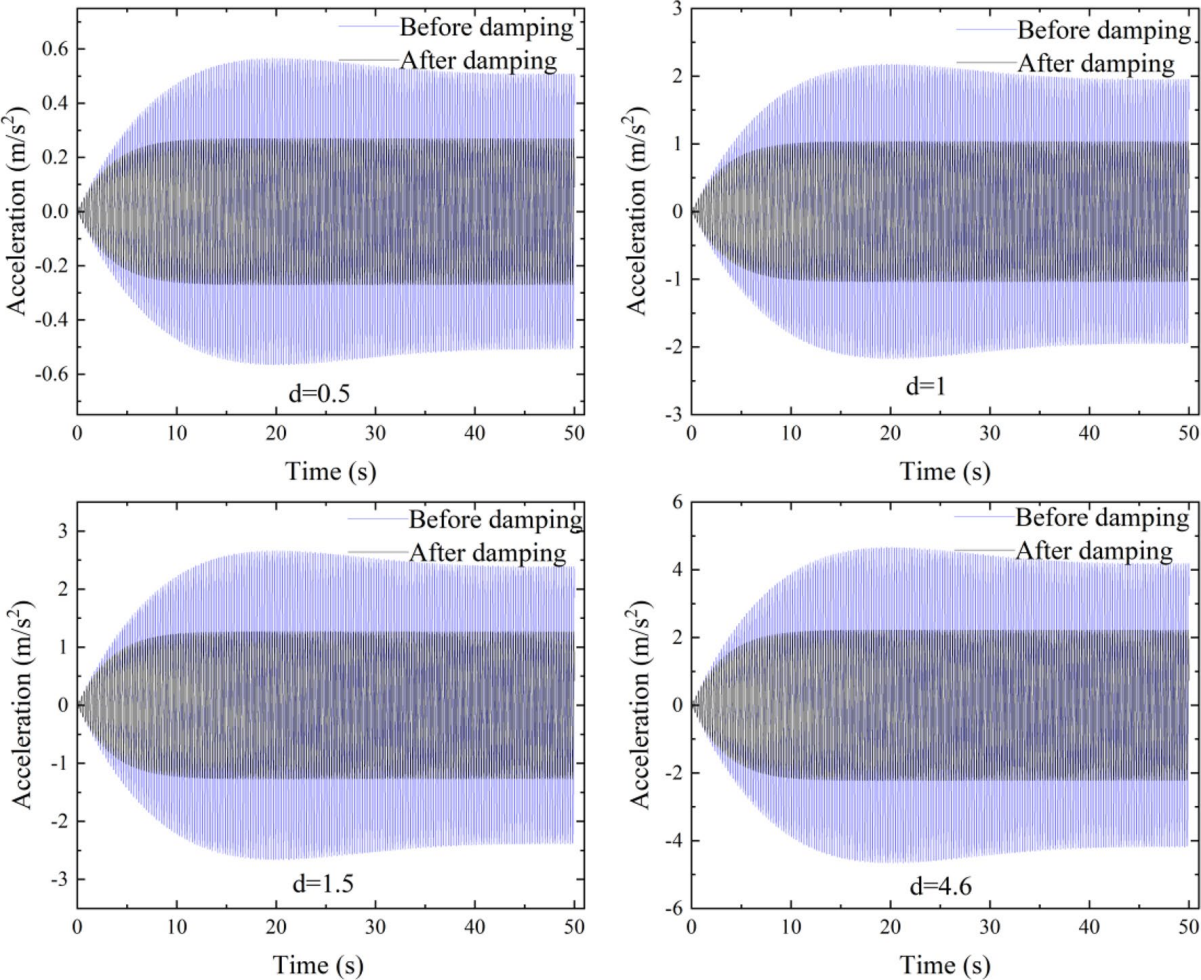
Comparison of the vibration damping effect of the TMD under working Condition 3.

The TMD is arranged at the point of maximum primary and secondary span acceleration response. The acceleration response time curves and acceleration response maxima before and after damping were compared for pedestrian densities of d = 0.5, 1, 1.5 and 4.6 persons/m^2^ under Condition 3, the resulting time curves are shown in Fig. 9, and the maximum acceleration and comfort ratings are shown in Table 8.

**Table 8:** Comparison of the vibration damping effect and comfort evaluation of the TMD after vibration damping under working Condition 3.

d (person/m^2^)	Maximum value acceleration (m/s^2^)			Comfort after damping
	Before damping	After damping	Vibration damping efficiency (%)	
0.5	0.566	0.271	52.12%	CL1
1	2.175	1.039	52.23%	CL3
1.5	2.664	1.273	52.21%	CL3
4.6	4.665	2.229	52.22%	CL3

The acceleration values in Table 8 are the average values of the absolute values of the forward and reverse accelerations. From Fig. 9 and Table 8, the acceleration response time curve shrinks significantly after setting the TMD, which indicates that the damping effect is obvious. The vibration damping efficiency can be more than 52%. The maximum acceleration response of 0.5 person/m^2^ after damping did not exceed 0.5 m/s^2^ and the CL1 comfort level was achieved. The comfort level of d = 1 person/m^2^ did not change, but the acceleration was close to the level of 1 m/s^2^. The comfort levels of 1.5 and 4.6 persons/m^2^ also decreased from CL4 to CL3. Therefore, the installation of a TMD has a significant effect on pedestrian bridge vibration reduction.

## Conclusions

1. Static testing shows that the articulated pier pedestrian bridge members do not exhibit obvious deformation. After the data summary, the maximum displacement of the three working conditions is 17.64, which does not exceed the specification limit, and the strength meets the design requirements.
2. When pedestrian loads are applied under the three abovementioned conditions, the maximum acceleration response occurs at the mid-span of the main span. The maximum acceleration response resulting from the simultaneous application of pedestrian loads on the primary and secondary spans can be considered the accumulation of the pedestrian loads applied directly on the full bridge.
3. The modal frequency and acceleration maxima are slightly larger in the pier rigid connection than in the pier articulation design.
4. When the crowd density of the articulated pier pedestrian bridge is greater than 0.5 people/m^2^, it is difficult for the comfort level to meet normal pedestrian use, which will cause pedestrian discomfort.
5. The installation of a TMD can effectively suppress the dynamic response under pedestrian loads, and the damping efficiency can reach 52%.

## Data Availability

The datasets used and/or analysed during the current study are available from the corresponding author upon reasonable request.
